# Cognitive Remediation in Bipolar (CRiB2): study protocol for a randomised controlled trial assessing efficacy and mechanisms of cognitive remediation therapy compared to treatment as usual

**DOI:** 10.1186/s12888-023-05327-1

**Published:** 2023-11-15

**Authors:** Dimosthenis Tsapekos, Rebecca Strawbridge, Matteo Cella, Kimberley Goldsmith, Michail Kalfas, Rosie H. Taylor, Samuel Swidzinski, Steven Marwaha, Libby Grey, Elizabeth Newton, Julie Shackleton, Paul J. Harrison, Michael Browning, Catherine Harmer, Hannah Hartland, David Cousins, Stephen Barton, Til Wykes, Allan H. Young

**Affiliations:** 1 Department of Psychological Medicine, Institute of Psychiatry, Psychology & Neuroscience, King’s College London, 103 Denmark Hill, London, SE5 8AZ UK; 2 Department of Psychology, Institute of Psychiatry, Psychology & Neuroscience, King’s College London, London, UK; 3 South London and Maudsley NHS Foundation Trust, Bethlem Royal Hospital, Beckenham, UK; 4 Department of Biostatistics & Health Informatics, Institute of Psychiatry, Psychology & Neuroscience, King’s College London, London, UK; 5 Institute for Mental Health, University of Birmingham, Edgbaston, Birmingham, UK; 6 Birmingham and Solihull Mental Health NHS Foundation Trust, Birmingham, UK; 7 Department of Psychiatry, University of Oxford, Oxford, UK; 8 Oxford Health NHS Foundation Trust, Oxford, UK; 9 Faculty of Medical Sciences, Translational and Clinical Research Institute, Newcastle University, Newcastle Upon Tyne, UK; 10 Cumbria, Northumberland, Tyne and Wear NHS Foundation Trust, Newcastle Upon Tyne, UK

**Keywords:** Bipolar disorder (BD), Cognitive remediation (CR), Randomised controlled trial (RCT), Efficacy, Mechanisms, Trial protocol

## Abstract

**Background:**

A substantial proportion of people with bipolar disorder (BD) experience persistent cognitive difficulties associated with impairments in psychosocial functioning and a poorer disorder course. Emerging evidence suggests that cognitive remediation (CR), a psychological intervention with established efficacy in people with schizophrenia, can also benefit people with BD. Following a proof-of-concept trial showing that CR is feasible and potentially beneficial for people with BD, we are conducting an adequately powered trial in euthymic people with BD to 1) determine whether an individual, therapist-supported, computerised CR can reduce cognitive difficulties and improve functional outcomes; and 2) explore how CR exerts its effects.

**Methods:**

CRiB2 is a two-arm, assessor-blind, multi-site, randomised controlled trial (RCT) comparing CR to treatment-as-usual (TAU). Participants are people with a diagnosis of BD, aged between 18 and 65, with no neurological or current substance use disorder, and currently euthymic. 250 participants will be recruited through primary, secondary, tertiary care, and the community. Participants will be block-randomised (1:1 ratio, stratified by site) to continue with their usual care (TAU) or receive a 12-week course of therapy and usual care (CR + TAU). The intervention comprises one-on-one CR sessions with a therapist supplemented with independent cognitive training for 30–40 h in total. Outcomes will be assessed at 13- and 25-weeks post-randomisation. Efficacy will be examined by intention-to-treat analyses estimating between-group differences in primary (i.e., psychosocial functioning at week 25 measured with the Functional Assessment Short Test) and secondary outcomes (i.e., measures of cognition, mood, patient-defined goals, and quality of life). Global cognition, metacognitive skills, affect fluctuation, and salivary cortisol levels will be evaluated as putative mechanisms of CR through mediation models.

**Discussion:**

This study will provide a robust evaluation of efficacy of CR in people with BD and examine the putative mechanisms by which this therapy works. The findings will contribute to determining the clinical utility of CR and potential mechanisms of action.

**Trial registration:**

Cognitive Remediation in Bipolar 2 (CRiB2): ISRCTN registry: https://www.isrctn.com/ISRCTN10362331. Registered 04 May 2022. Overall trial status: Ongoing; Recruitment status: Recruiting.

## Background

Bipolar disorder (BD) is a chronic and recurrent mental health condition characterised by the presence of manic, hypomanic, and depressive episodes which are interspersed with phases of euthymia [1]. For disorders in the wider BD spectrum, the yearly prevalence is around 2% [2]. It is classified as one of the leading causes of disability globally, accounting both for direct medical costs and indirect societal burden, such as loss of productivity and economic growth [3, 4]. In the UK, BD accounts for 1.5% of the total illness burden [5], while the annual direct cost of managing BD has been estimated at £343 million [6].

A feature of BD that has recently attracted significant research and clinical interest is cognitive impairment. Moderate-to-severe deficits in multiple cognitive domains, such as attention and processing speed, verbal memory, and executive functions, have been reported for 40–60% of the patients [7, 8]. These cognitive difficulties often persist during periods of remission [9], affect episode recurrence [10], and contribute to later functional disability, including occupational capacity [11, 12]. As the ultimate goal of clinical practice is functional recovery and improved quality of life [13], the relevance of cognitive difficulties to psychosocial difficulties highlighted the need for treatments in this area. However, there is currently limited availability of evidence-based interventions with direct and durable effects on cognition for BD [14].

Pharmacological compounds previously examined as potential pro-cognitive agents showed limited evidence of clinical efficacy, particularly for overall functioning [15]. A candidate treatment is Cognitive Remediation (CR), an evidence-based psychological intervention targeting cognition to improve functioning, supported by compelling evidence for people with schizophrenia [16, 17] and recommended as a first-line treatment of cognitive impairment in people with psychosis [18].

Given the similarities in the cognitive profile of schizophrenia and BD [19], CR was identified as a treatment with the potential to achieve comparable effects on cognition and functioning for people with BD [20]. However, findings from initial open-label studies, although encouraging, were uncontrolled and subject to limitations, while the first randomised trials reported inconsistent results [21]. Low effect sizes might be attributed to methodological and intervention characteristics, such as small effects being associated with group interventions on cognition [22] and those without a substantive therapist component reporting modest effect sizes on functioning [23]. Patient characteristics may also contribute to inconsistent effects [24–27].

Our group recently conducted the Cognitive Remediation in Bipolar (CRiB) study: a feasibility and randomized pilot trial evaluating a 12-week, therapist-supported, computerised, individualised CR programme [28]. This manualised approach (Computerised Interactive Remediation of Cognition and Thinking Skills; CIRCuiTS™) combined intensive cognitive training with strategy use, metacognitive skill development, and transfer of cognitive skills to everyday life [29, 30]. No screening for cognitive difficulties was applied at study entry. The intervention was feasible to deliver (76% completing > 20 h therapy) and highly acceptable (95% perceived improvement) to euthymic people with BD. Compared to treatment-as-usual (TAU), those who received CR showed medium-to-large cognitive and functional improvements after the intervention that were maintained 12 weeks later [31]. A secondary analysis indicated that post-treatment cognitive improvement was independent of participants’ cognitive level and other pre-intervention characteristics [32].

Although promising, CRiB was underpowered (*N* = 80) to answer the question of efficacy. A recent meta-analysis combining CR studies across affective disorders reported that only 4 out of 22 studies focused exclusively on BD and none had an adequate sample size [33]. This meta-analysis found that CR improved several cognitive domains with small-to-medium effect sizes but had no effect on functional outcomes, such as community and occupational functioning. The authors proposed that future research should focus on how therapy-related cognitive improvements can be generalised to functional outcomes [33], in line with methodological recommendations for cognition trials by International Society for Bipolar Disorders (ISBD) [34].

Given that there is a putative signal for CR in people with BD, we need to know if the signal can be identified by biological and non-biological markers. These potential markers could help to tailor treatments and adjust the therapy ingredients. Biological markers may include cortisol, a hormone involved in the regulation of cognitive processes, with evidence supporting an association between cortisol level changes and cognitive functioning for people with mood disorders [35]. CR potentially exerts its cognitive effect by influencing cortisol secretion. A caveat is the role of dehydroepiandrosterone (DHEA), a hormone that can mitigate the cortisol effects [36], so the ratio of cortisol to DHEA may be more informative of “functional” cortisol levels. For people with BD, a higher cortisol/DHEA ratio has been associated with poorer performance in executive functioning [37].

The CR model suggests that an increase in cognition will fuel functional improvement and an exploratory analysis from CRiB study supports this contention as post-treatment cognitive gains accounted for more than one third (35%) of the treatment effect on psychosocial functioning at the 6-month follow-up [38]. However, this still leaves a substantial proportion of the treatment effect unaccounted for. Our CR approach directly targets metacognitive skills, such as the understanding of own strengths or limitations and the ability to apply strategies. Hence, improved metacognition may be one of the factors enabling success in functional tasks and explaining the effect of CR on functioning [39]. Another candidate mechanism in people with BD is the regulation of affective instability, a characteristic feature of BD associated with adverse outcomes, such as functional impairment [40]. Enhancing cognitive control through CR may lead to reduced affect fluctuation, thus improving psychosocial functioning.

In the last decade, there has been substantial progress in understanding cognitive impairment in BD and addressing it as an independent treatment target. Currently, the field is at the stage where robustly designed and appropriately powered clinical trials are required to provide definitive evidence on the efficacy of CR on cognition and functioning, as well as to examine putative mechanisms that may drive these effects. Potential moderators of response also remain to be explored. This may have substantial implications for clinical practice and the quality of care offered to patients with BD, as well as reducing the costs of care.

### Aims and objectives

The overarching aim is to determine whether a 12-week, individual, therapist-supported, metacognition-informed, computerised CR intervention provides substantial and durable benefits for the daily lives of euthymic patients with BD. To achieve this, we will assess the efficacy and evaluate putative treatment mechanisms of CR.

#### Primary objective

- To assess the efficacy of CR added to TAU compared to TAU alone (CR vs. TAU) for improving psychosocial functioning in people with BD at 25 weeks after randomisation.

#### Secondary objectives

- To assess the efficacy of CR vs. TAU for the improvement of psychosocial functioning at 13 weeks after randomisation, and the improvement of cognition (global cognition and individual domains), subjective cognitive complaints, severity of mood symptoms, sleep quality, patient-defined goal attainment, and health-related quality of life at both 13 and 25 weeks after randomisation.
- To investigate cortisol as a putative treatment mechanism of CR effects on cognition by analysing the association between post-treatment change in cortisol secretion (week 13 levels, adjusted for week 0) and subsequent change in global cognition for CR vs. TAU at 25 weeks.
- To investigate global cognition, metacognitive skills, and affect fluctuation as putative CR treatment mechanisms for functioning by analysing the association between post-treatment change in these outcomes (week 13 scores, adjusted for week 0) and subsequent change in psychosocial functioning for CR vs. TAU at 25 weeks.

The moderating effect of pre-randomisation patient characteristics on the CR treatment response, as well as other tertiary and exploratory outcomes, will be explored in a separate publication.

## Methods

### Trial design

**Fig. 1:**
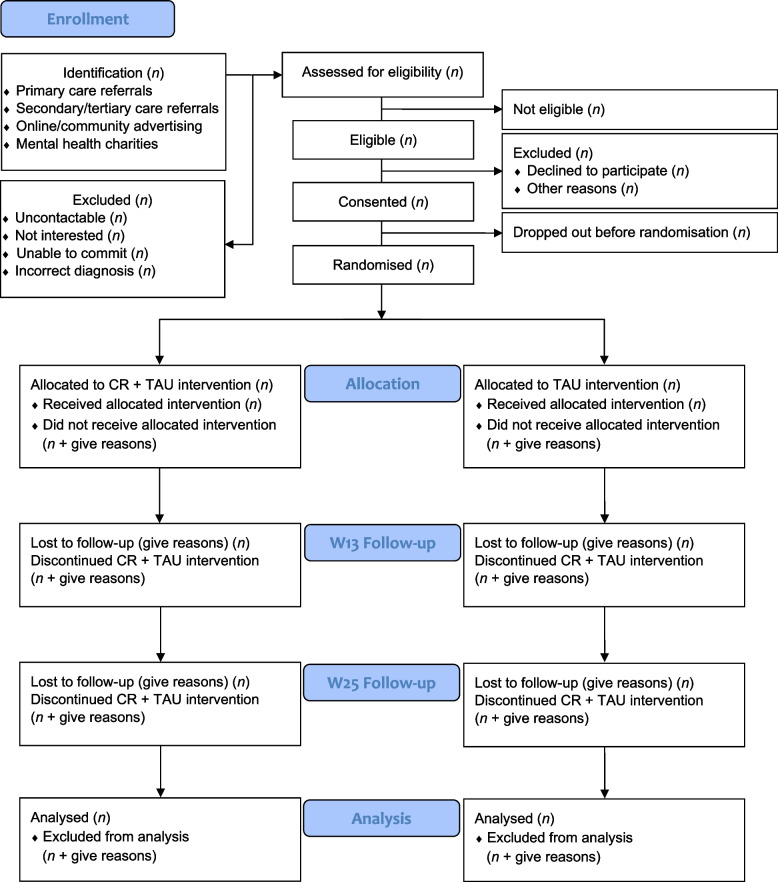
CONSORT flow diagram of study procedures

This is a two-arm parallel group, assessor-blind, randomised controlled trial (RCT) comprising a 12-week intervention period followed by a 12-week follow-up period. Assessments will be conducted at week 0 (W0; baseline) and then at week 13 (W13; post) and 25 (W25; follow-up) post-randomisation. Two hundred and fifty participants (*N* = 250) will be randomised in a 1:1 ratio to either CR plus TAU (*n* = 125) or TAU alone (*n* = 125). See Fig. 1 for the study flowchart.

### Setting

This is a multi-centre trial taking place in four sites across the UK: London, Oxford, Birmingham, and Newcastle. Assessments are conducted at a Clinical Research Facility (CRF) or an equivalent outpatient facility. Therapy sessions take place at the same facilities or online via video call. All study procedures are conducted in quiet, private rooms with appropriate setup for cognitive testing or therapy delivery.

### Target population

The study will include patients with BD from different care levels and settings. Participants can only be included if meeting all the inclusion criteria and not violating any exclusion criteria.

#### Inclusion criteria

- Adults aged 18–65 years;
- DSM-5 diagnosis of bipolar type I or II (validated using the Mini International Neuropsychiatric Interview [MINI] 7);
- Euthymic according to the Newcastle Euthymia Protocol, requiring a score of less than 8 on both the Hamilton Rating Scale for Depression (HAMD) and Young Mania Rating Scale (YMRS) covering the entire month prior to inclusion;
- Ability to use a computerised device (defined as having used a computer, a tablet, or a smartphone at least once in the prior 4 weeks independently).

#### Exclusion criteria

- Comorbid alcohol/substance use diagnosis in the past 12 months (assessed using the MINI 7);
- Current risk of suicide (assessed using the MINI 5);
- Indications of cognitive decline (assessed using the Montreal Cognitive Assessment – Telephone version [MoCA-T]) or impairing organic neurological disorder (assessed using patient-report and checked with a medical practitioner);
- Having an IQ < 80 estimated by the Test of Premorbid Functioning (TOPF);
- Having undertaken a manualised CR therapy any time in the past;
- Inability to communicate fluently in English (defined as ability to read and understand the participant information sheet – at a similar reading level as the CR programme – and to communicate with the researcher throughout screening procedures);
- Currently undergoing a formal psychological therapy or specifically planning changes to treatment (medication change or initiation of a new therapy) over the coming 6 months (trial period);
- Not being registered with a primary healthcare professional in the UK (i.e., a General Practice; GP) or unwillingness to provide their GP contact details;
- Inability to travel to one of the research sites on a regular basis over 25 weeks;
- Inability to provide informed consent to participate, for any other reason.

Where there is any doubt about the validity of the BD diagnosis, euthymia (as defined above), current suicide risk, or impairing neurological disorder, each potential participant’s assessments will be validated with a practising psychiatrist collaborating with the study team (Chief Investigator [CI], Principal Investigators [PI], clinical leads, or other clinicians within the participating research teams).

#### Withdrawal

Participants can be withdrawn in the event of BD symptom exacerbation, or adverse events associated with the trial or therapy procedures. All participants are free to withdraw from the study at any time, without providing a reason. Similarly, therapy can be discontinued at any point, if a participant withdraws from the trial or decides they no longer wish to continue the intervention. If a participant withdraws from the intervention only, all efforts will be made to continue obtaining follow-up data. Details of any withdrawals and, if possible, reasons for dropping out will be recorded in withdrawal forms.

### Intervention

Participants in the treatment arm will receive a 12-week course of CR therapy using the online software CIRCuiTS™ (www.circuitstherapy.com). CIRCuiTS™ is a manualised CR approach, developed according to the key principles of CR (e.g., errorless learning, scaffolding, positive feedback, strategy development) [41]. CIRCuiTS™ employs rigorous cognitive training with computerised tasks, emphasises the identification and implementation of useful strategies, supports the development of metacognitive skills, as well as the transfer of cognitive skills and strategies to daily life activities.

CR will be delivered to participants over the 12-week intervention period. Sessions are one-on-one 1-h with the therapist, either in person or remotely (i.e., video call). Additionally, all participants in therapy have access to CIRCuiTS™ for independent sessions, with practice tasks agreed with the therapist. The target for therapy engagement is 2–3 hourly sessions per week aiming at a total of 30–40 sessions over 12 weeks.

#### Therapy training

CR will be delivered by postgraduate level therapists who have completed online training (approximately 25 h) supplemented by a period of supervised practice using CIRCuiTS™ with people with BD. Every therapist will work with at least one training client before undertaking any trial responsibilities.

#### Therapy supervision

All therapists will receive supervision from an experienced clinical psychologist (MC). Supervision will be provided weekly in a group format (including therapists from all sites) to discuss participants in therapy, case formulations, adherence and implementation challenges, and best practice to facilitate transfer. A supervision log will record the content, duration and input provided in each supervision session. If needed, group supervision sessions will be supplemented by individual sessions to discuss training needs, personal issues, and professional development.

#### Treatment fidelity

Fidelity to the therapy approach and the core principles of CR with CIRCuiTS™ will be initially ensured through the provision of similar training across trial therapists and will be continuously evaluated through supervision with additional information provided through the software engagement and use of therapy-specific processes.

#### Treatment adherence and retention

Following evidence from a secondary analysis of our feasibility study [42], 20 h was defined as the minimum therapy dose. This includes time spent in face-to-face or remote therapist-supported sessions and time spent on independent training. Strategies employed for engaging participants with the intervention involve planning sessions tailored to participant preferences and schedule and sending text reminders ahead of sessions. Individual therapy logs will be used to monitor and record treatment adherence.

Participants will be offered feedback on their baseline neuropsychological test performance after the W25 assessment. In addition, participants allocated to the TAU only group, will be offered access to CIRCuiTS™, as well as a one-off session with a therapist, after the W25 assessment. Each participant will be compensated with £20 for each study assessment, while travel expenses for public transportation or mileage will be reimbursed for all participants.

#### Treatment as usual

Participants in both trial arms will continue to receive their standard care without any interference from the trial team. Pharmacological and non-pharmacological treatments, as well as any use of healthcare services, will be monitored and recorded for all participants at each post-randomisation visit.

### Recruitment

Participants will be recruited from secondary/tertiary care services within the local NHS Trust of each site, primary care services (i.e., GP surgeries) in collaboration with the National Institute for Health and Care Research (NIHR) Clinical Research Network, and the community through online/offline advertising and in collaboration with mental health charities (e.g., Bipolar UK). Some Trusts also allow the identification of patients who have consented to be contacted for participation in research projects through electronic medical records.

### Procedure

Following potential participation through clinician referral or direct expression of interest, potential participants will be provided with the Participant Information Sheet (PIS) and time to ask questions and consider whether they wish to participate or not (i.e., at least 24 h prior to initial screening). Two screening sessions by phone will be carried out over one month to ensure euthymia. Eligible participants will be provided with a provisional in-person appointment to complete and sign a written Informed Consent Form (ICF), then the baseline assessment will be conducted, and the participant will be randomly allocated to the treatment or control group. Post-randomisation assessments will be conducted at W13 and W25. The journey of each participant through the trial is outlined in Fig. 1.

### Randomisation and blinding

Participants will be randomly allocated in a 1:1 ratio to one of the two treatment arms (CR + TAU or TAU only) on the same day of the baseline assessment, using a web-based randomisation system which employs randomly varying block sizes, stratified by site. This randomisation system is managed by the King’s Clinical Trials Unit (KCTU). The details needed for randomisation (i.e., study site, month/year of birth, initials, and unique patient identity number) will be held in a dedicated database with only the trial manager having access to the randomisation system. The trial manager will inform participants about group allocation by telephone within 1–2 working days.

While it is not possible to blind therapists and participants, researchers conducting post-randomisation assessments will remain blind to group allocation. Participants will be asked not to disclose their allocation but, if a researcher is informed, they will be replaced by a different assessor for the remaining assessments. This procedure was successfully implemented in the CRiB study [31]. Blinding will be maintained until the last participant completes the follow-up assessment. Data will be collected from each assessor to test the robustness and maintenance of blinding. At both W13 and W25, assessors will be asked by the trial manager whether they have been explicitly unblinded for this participant. Ratio (%) of unblinding will be estimated and reported for both the treatment and the control group.

### Sample size and power

Our sample size estimation was based on the CRIB study findings: a standardised effect size of 0.49 on the Functional Assessment Short Test (FAST) at W25, a correlation of 0.7 between baseline and follow-up, and an intraclass correlation coefficient (ICC) of 0.02 [31]. Accounting for the effect of therapist clustering, precision gain from one pre and two post-randomisation repeated measures, and an attrition rate of 15%, a power calculation suggested that including 125 participants per arm gives 90% power to detect an effect size of 0.37, which is smaller than the CRIB study reported effect size (i.e., it is a conservative estimation, due to a propensity for small samples to overestimate treatment effects).

### Data collection and management

The measures collected at each time-point are presented in Table 1. If a participant withdraws from the study, the data already collected will be kept and included the analysis. Retention rates will be monitored.

**Table 1:** Schedule of enrolment, interventions, and assessments

		**Trial time-points**						
**Procedures/Measures**	**Completed by**	**Screen A (W-2)**	**Screen B (preW0)**	**Baseline (W0)**	**Intervention (W1-12)**	**Post (W13)**	**Follow-up (W25)**	**All (W1-25)**
*Administrative*								
Eligibility assessment	R	x	x					
Informed consent	R			x				
Randomisation	TM			x				
CR delivery	T				x			
Blinding	R					x	x	
*Eligibility*								
Patient information	R	x						
MINI 7	R	x		x ^a^				
TOPF / MoCA-T	R	x						
HAMD / YMRS	R	x	x	x		x	x	
*Interview-based*								
Sociodemographic	R			x		x	x	
Illness history	R			x				
Service use	R			x		x	x	
*Baseline only*								
HAMA	R			x				
CTQ / MEQ / MSI-BPD	P			x				
*Efficacy outcomes*								
Cognitive battery (Hotel test, WMS-IV ^b^, WAIS-IV ^b^, TMT, FAS)	R			x		x	x	
FAST / GAS / EQ5D-3L	R			x		x	x	
PDQ / PSQI	P			x		x	x	
*Mechanistic outcomes*								
Saliva sample	P			x		x		
PANAS	P			x		x		
MAI / Torres	P / R			x		x	x	
*Therapy-related* ^c^								
Therapy log	T				x			
WAI-SR	P & T				x (W3, W12)			
CR satisfaction	TM					x		
*Monitoring*								
Adverse events	R & T			x		x	x	x
Concomitant Tx	R			x		x	x	
Mood monitoring	P							x

*CR* Cognitive Remediation Therapy, *CTQ* Childhood Trauma Questionnaire, *EQ5D-3L* EuroQoL-5 Dimensions – 3 Levels, *FAS* F-A-S letters verbal fluency test, *FAST* Functioning Assessment Short Test, *GAS* Goal Attainment Scale, *HAMA* Hamilton Anxiety Rating Scale, *HAMD* Hamilton Depression Rating Scale, *MAI* Metacognitive Awareness Inventory, *MEQ* Morningness-Eveningness Questionnaire, *MINI 7* Mini International Neuropsychiatric Interview for DSM-5, *MoCA-T* Montreal Cognitive Assessment-Telephone version, *MSI-BPD* MacLean Screening Instrument for Borderline Personality Disorder, *MVAS* Maudsley Visual Analogue Scale, *PANAS* Positive and Negative Affect Schedule, *PDQ* Perceived Deficits Questionnaire, *PSQI* Pittsburgh Sleep Quality Index, *TMT* Trail Making Test, *TOPF* Test of Premorbid Functioning, *Tx* Treatment, *W* Week, *WAIS* Wechsler Adult Intelligence Scale, 4^th^ edition, *WAI-SR* Working Alliance Inventory-Short Revised, *WMS-IV* Wechsler Memory Scale, 4^th^ edition, *YMRS* Young Mania Rating Scale

*Roles:* P: Participant; R: Researcher; T: Therapist; TM: Trial Manager

^a^MINI comorbidity data not required prior to inclusion (e.g., anxiety disorders) will be assessed at W0 instead of W-2

^b^Preselected tests rather than whole scales: WMS-IV: Verbal Paired Associates I & II; WAIS-IV: Digit Span & Coding

^c^Procedures/measures referring only to participants allocated to the intervention group

Source data will be collected on paper and electronically and entered by trained authorised team members, typically within 14 days of data collection, onto an online electronic data capture (EDC) using the InferMed MACRO 4 system, created and maintained by the KCTU. EDC access will be strictly restricted through user-specific passwords to the authorised research team members. The trial manager will conduct random checks for data correctness and completeness. Following the final check, data will be formally locked for analysis. KCTU will provide a copy of the final exported dataset to the CI and the trial statisticians. The overall custodian for the trial data will be the CI.

### Measures

All study measures are presented in Table 1.

#### Baseline

1. *Socio-demographic* characteristics (e.g., gender, ethnicity, height, weight, education, employment).
2. *Illness-history/clinical* characteristics (e.g., diagnostic type, previous episodes, history of psychosis).
3. *Service use* (e.g., past/current pharmacological & non-pharmacological treatments, hospitalisations).

#### Primary outcome

1. *Psychosocial functioning*: The FAST [43] will be used to assess levels of functioning. The FAST consists of 24 items (rated on 0-3 scale), which assess 6 areas of daily life functioning: autonomy (ability to doing this alone and making own decisions); occupational functioning (ability to work); cognitive functioning (patient’s cognitive abilities); financial issues (ability to manage finances); interpersonal relationships (social relationships with family and friends); and leisure time (engaging in hobbies and physical activity). Higher scores indicate greater difficulties. The FAST was specifically designed to examine difficulties experienced in BD and is recommended for clinical research by the ISBD [34].

#### Secondary outcomes

1. *Cognition*: Composite score and individual domain scores for attention and processing speed (Digit-symbol Coding test from the Wechsler Adult Intelligence Scale 4th edition [44]; Trail Making Test A [TMT] [45]), working memory (Digit Span test from the Wechsler Adult Intelligence Scale 4th edition) [44], verbal learning and memory (Verbal Paired Associates) [46], and executive functioning (Hotel Test [47]; FAS letter fluency test [48]; TMT B [45]). The global cognition composite score will be calculated as per [31].
2. *Mood symptoms* using HAMD [49] for depression and YMRS [50] for hypomania/mania.
3. *Subjective cognitive complaints* using the Perceived Deficits Questionnaire – Depression (PDQ-D) [51].
4. Attainment of patient-defined goals using the Goal Attainment Scaling (GAS) [52].
5. *Sleep quality* the using Pittsburgh Sleep Quality Index (PSQI) [53].
6. *Health related quality of life* using Euro Quality of Life – 5 Dimension – 3 Levels (EQ-5D-3L) [54].

#### Mechanistic outcomes

1. *Global cognition* using a composite score of individual cognitive tests*.*
2. *Saliva samples* to estimate Cortisol Awakening Response (CAR), full-day cortisol secretion levels, and the ratio of cortisol to DHEA [36, 55]
3. *Metacognition* using the Metacognitive Awareness Inventory (MAI) [56] and the Torres’ ratings for metacognitive knowledge/experience [57].
4. Positive and negative *affect fluctuation* using the Positive and Negative Affect Schedule – Short Form (PANAS-SF) [58].

Saliva samples will be self-collected by participants on one day within the three days following the assessment at W0 and W13. Six samples will be collected over the day using the cotton swab method: 0, 15, 30, 45, and 60 min after awakening, and then at 8pm [59]. Self-collection packs containing saliva tubes will be sent back to the London site, using Royal Mail Tracked delivery.

PANAS will be completed by participants twice daily for seven consecutive days following the assessment at W0 and W13, using a secure web platform (Qualtrics; https://www.qualtrics.com/). Variability between daily mood ratings throughout the week will subsequently be computed to measure daily affect fluctuation, split into positive and negative volatility (the measure of interest; defined as the change in the mean of the mood ratings between ratings) and positive and negative noise (defined as variability in affect that does not persist between ratings) [60]. Participants will be prompted to complete these ratings with automated email reminders.

#### Tertiary outcomes

1. Pre-randomisation variables: *Premorbid IQ* using the Test of Premorbid Functioning (TOPF) [61]; *childhood trauma* using the Childhood Trauma Questionnaire – Short Form (CTQ-SF) [62]; *anxiety* using the Hamilton Rating Scale for Anxiety (HAMA) [63]; *chronotype* using the Morningness-Eveningness Questionnaire (MEQ) [64]; and *traits of borderline personality disorder* using the MacLean Screening Instrument for Borderline Personality Disorder (MSI-BPD) [65].
2. Therapy-related variables: *Session attendance* using the individual therapy log for each participant; *treatment adherence* using the pre-defined threshold of ≥ 20 therapy hours; and *therapeutic alliance* for both the therapist and the client using the Working Alliance Inventory-Short Revised (WAI-SR) [66].

#### Other measures

At screening and baseline, participants will undergo a diagnostic interview for common mental disorders (i.e., major depressive episode, manic and hypomanic episode, alcohol use disorder, substance use disorder) using the MINI 7 [67], whilst suicidality will be assessed using MINI 5 [67]. A brief screening for indications of cognitive decline associated with organic/neurological condition will be also conducted using the Montreal Cognitive Assessment – Telephone version (MoCA-T) [68]. Participants in the intervention group will also complete a CR satisfaction questionnaire [69]. Finally, all participants will be asked to complete brief mood assessments on a weekly basis from W1 to W24 using the Maudsley Visual Analogue Scale (MVAS) which will be only used to monitor mood and wellbeing of participants during the trial [70].

### Statistics

We provide a brief summary here, however a detailed statistical analysis plan (SAP) will be developed by the trial statisticians and approved by the trial team and the oversight committees. The main analysis will adopt the intention-to-treat principle and will be conducted after data collection has been completed, the data cleaned, and the database locked.

To ascertain the differences in primary and secondary outcome measures between participants randomised to CR + TAU and TAU alone, mean differences between the groups (and their 95% confidence intervals) in the primary (FAST score at W25) and secondary outcomes will be estimated using mixed-effects linear regression models with the W13 and W25 measures of the outcome in question as dependent variables. Models will include a random intercept for participants, time, and time-by-treatment terms (to allow for extraction of mean differences between treatment groups at different time points), baseline measure of the outcome and site as pre-specified covariates. Standardised effect sizes will be computed, by dividing the estimated group difference by the pooled baseline standard deviation of the measure, to quantify the effect of treatment on primary and secondary outcomes. We will assess whether baseline characteristics are predictive of missing data and include these in the analysis models using maximum likelihood methods under the missing-at-random assumption to account for missing data.

Treatment effect mediation via mechanistic measures will be assessed either using structural equation modelling or causal mediation analysis (e.g., *paramed* command in Stata), adjusting for baseline measures of the mediator and outcome, site, age, gender, and other potential mediator-outcome confounders [71]. We will use the W13 measures of the putative mediator variables and W25 measure of the outcome in mediation models. We will explore whether treatment effects on the global cognition measure are mediated via cortisol measures and whether treatment effects on the FAST are mediated via global cognition, metacognition, and/or PANAS affect fluctuation.

### Trial oversight

The trial will be overseen by the Trial Management Group (TMG), as well as two fully or partially independent committees, the Data Monitoring and Ethics Committee (DMEC) and the Trial Steering Committee (TSC). Both the TMG and the DMEC/TSC will provide overall supervision of the trial and ensure that it is conducted according to the Good Clinical Practice (GCP) guidelines. The DMEC will monitor data collection, including data quality and safety information. The TSC will be monitoring the overall progress of the trial and ensure that study procedures are conducted in adherence to the protocol. The study investigators will provide trial-related monitoring, audits, and reviews from the Sponsor or the Research Ethics Committee (REC).

### Service user involvement

People with lived experience were involved with both the conception and the development of this project, as well as the conduct of the trial. Trial design was reviewed by the Service User Advisory Group (SUAG) of the NIHR Maudsley Biomedical Research Centre (BRC) which found CRiB2 to address a clinical area of need. Based on their recommendation we decided not to exclude people with a co-morbid personality disorder diagnosis to make the sample more representative. The NIHR Maudsley BRC Feasibility and Acceptability Support Team for Researchers (FAST-R) also reviewed the trial plan and relevant study information. Based on their feedback, we adopted improvement of psychosocial functioning as the trial’s primary endpoint to reflect the prioritisation of functional recovery as an outcome. We also reduced technical language in participant-facing documentation (i.e., information sheet and consent form). In collaboration with Bipolar UK, a focus group was held prior to protocol finalisation which reviewed study procedures and made suggestions to improve trial procedures from a patient perspective (i.e., preventing participant over-burden, improving appointment scheduling, debriefing, and providing feedback).

Two service users are part of our TSC and provide regular feedback on the study conduct and issues arising with its progress. For example, their input informed our procedures for remote data collection (i.e., saliva samples/PANAS) with the aim of making it as simple and non-intrusive for study participants as possible. Service users also offer advice on how to approach potential participants and improve our recruitment strategies. A researcher with BD diagnosis joined the study team at the application stage to support the project from an expert-by-experience perspective. We are in the process of developing an advisory group of service users with experience of BD and representation at each site who will provide advice throughout the trial. They will be involved in data analysis through advisory workshops and dissemination, including as authors of our papers and plain English summaries for the BD community and the wider public.

### Safety monitoring

Potential adverse events (AE), serious adverse events (SAE), adverse reactions (AR), unexpected adverse reactions (UAR), serious adverse reactions (SAR) and suspected unexpected serious adverse reactions (SUSAR) will be monitored and recorded throughout the trial using the AE log in the participant case report form. In addition, therapists will be collecting AE throughout the intervention.

Investigators will assess whether the AE may be related to study participation or therapy and will also assess the severity of the event. Classification of AE as SAE will be based on whether the event: a) results in death, b) is life-threatening, c) requires hospitalisation or prolongation of hospitalisation (not including one for pre-existing condition), d) results in persistent or significant disability, and e) results in an important medical event (IME). All AE will be also reviewed by the DMEC to establish the relatedness to the trial or the intervention and whether these can be classified as SAE/AR/UAR/SAR/SUSAR. All AR and UAR will be reported within 24 h to the CI, as well as be considered in committee meetings. All SAE and SAR will be reported within 24 h by the CI to the Sponsor. Only SUSARs will be reported within 7 days by the CI to the REC for review.

Although we do not anticipate any SARs or SUSARs, participants presenting any such reactions will be withdrawn from the CR intervention and, if not already done by the participant, these reactions will be brought to the attention of their named healthcare professional. AE of any category that have not resolved by the end of the trial, or that have not resolved upon discontinuation of the subject’s participation in the trial, will be followed until it resolves, stabilises, returns to baseline, or cannot be attributed to trial participation or to factors related to the trial.

### Stopping rules

There are no plans for a formal interim analysis or formal stopping rules for the trial. The trial may be prematurely discontinued for safety reasons, lack of recruitment or other concerns regarding trial data.

### Ethics and dissemination

#### Research ethics approval

Ethical approval has been granted by the London – Bromley REC in Spring 2022 (22/LO/0210). King’s College London and South London and Maudsley NHS Foundation Trust have jointly sponsored the trial.

#### Confidentiality

We will adhere to NHS and Research Governance Framework confidentiality practices, with data being pseudonymised when recorded in the database: unique personal identification number (PIN) assigned to each participant along with their initials and month/year of birth. Identifiable information will be stored on a password-protected spreadsheet within the KCL server in accordance with the General Data Protection Regulation (GDPR) and King’s Research Governance guidelines.

#### Dissemination policy

The results of the study will be presented at international scientific conferences and reported in peer-reviewed scientific journals. A primary publication will include all primary and secondary outcomes as per the published protocol, and further publications will explore tertiary outcomes and other mechanistic components of the intervention. We will also provide lay summaries of the findings to charities and the public.

## Discussion

The clinical management of BD presents with a significant gap in addressing cognitive and functional difficulties. Research interest in interventions targeting cognition has only recently emerged. Our group previously demonstrated that CIRCuiTS™ is feasible to deliver, highly acceptable, and potentially beneficial for cognitive skills which translate to functional improvement. This protocol outlines the rationale and design of a robust and appropriately powered RCT aiming to determine the efficacy of CIRCuiTS™ on psychosocial functioning and cognition, and examine putative biological and psychological mechanisms underlying treatment effects in euthymic patients with BD.

### Strengths and limitations

A strength of the present study is the rigorous methodological design, including: a large sample size providing adequate power for the intended analysis; a high-quality randomisation system and a secure electronic data capture system designed and managed by the KCTU; the selection of validated measures; multiple procedures to maintain assessor blinding and verify blinding success; a pre-specified analysis plan adopting the intention-to-treat principle; training and ongoing support for outcome assessors; and a manualised intervention delivered by trained therapist under regular supervision. Methodological rigour will be also assured by two independent committees, the DMEC (fully independent) and the TSC (> 75% independent).

There are also limitations that need to be considered when evaluating the impact and implications of this trial. First, the absence of an active control group limits our ability to account for non-specific therapeutic effects. This could also increase the attrition rate in the control group. However, those individuals will have access to CIRCuiTS™ at the end of the trial. CR would be a distinct addition to the standard treatment offered to patients with BD which justifies the use of TAU as a comparator. No screening for cognitive or functional impairment is implemented at study entry to increase the generalisability of our findings but this may introduce ceiling effects for these outcomes, although that was not the case in our feasibility study. Recruiting participants from different routes and levels of care could benefit the representativeness of our sample, but it may increase heterogeneity in our findings, but we may be able to account for that through moderation analysis. The flexibility of CR delivery (in-person, online, homework), whilst beneficial for engagement, may also increase heterogeneity. Only euthymic participants will be recruited and, although this has the potential to create recruitment challenges, this was not the case in our previous study.

### Potential implications

We anticipate that findings from this trial will provide robust evidence about the efficacy of CR after and beyond the intervention period. This may accelerate its integration for wider use in clinical practice. If CR is found to be efficacious, this can prompt the conduct of further clinical trials examining the effectiveness of CR and how it can be clinically implemented in efficient and cost-effective ways. Identifying treatment mechanisms will improve our understanding of appropriate engagement with therapeutic targets driving cognitive or functional improvement and inform future mechanistic studies. A future schedule of studies might also examine research questions on health economics, time, staff, and training resources, combination strategies, and differential treatment response to improve the personalisation of CR. In the long-term, implementation of CR in clinical services may significantly improve the overall quality of care provided to people with BD, facilitate functional recovery, and enhance quality of life. This may contribute to reductions in service use requirements (e.g., inpatient admissions, secondary/tertiary settings) and, subsequently, lead to substantive economic benefits through the reduction of both direct and indirect costs associated with BD.

### Trial status and engagement

King’s College London and South London and Maudsley NHS Foundation Trust agreed to provide sponsorship for this trial. A favourable ethical opinion was granted by the London – Bromley REC (Reference 22/LO/0210). The trial is ongoing. Recruitment commenced in July 2022 and will continue until at least June 2024. The publication of our protocol aims to maximise reproducibility and transparency of the CRiB2 trial.

Study information is available in the CRiB2 webpage within the King’s College London website. Study findings will be shared with our participants through a newsletter, while we also plan to hold a “celebration event” at the end of the trial where we will invite all researchers and other stakeholders, patient representatives and participants across all sites. This will contain a presentation and an open discussion on trial outcomes and next steps, but also taking feedback from different contributors and organising smaller group exercises (“mini focus groups”) to develop a set of recommendations for future activities.

## Data Availability

Not applicable (NA); this protocol does not contain any data.

## Abbreviations

AE: Adverse Event
AR: Adverse Reaction
BD: Bipolar disorder
BRC: Biomedical Research Centre
CAR: Cortisol Awakening Response
CI: Chief Investigator
CIRCuiTS: Computerised Interactive Remediation of Cognition - Interactive Training for Schizophrenia
CR: Cognitive Remediation
CRF: Clinical Research Facility
CRiB: Cognitive Remediation in Bipolar
CRIS: Clinical Record Interactive Search
CTQ-SF: Childhood Trauma Questionnaire
DHEA: Dehydroepiandrosterone
DMEC: Data Monitoring and Ethics Committee
EDC: Electronic Data Capture
EQ-5D-3L: EuroQoL-5 Dimensions - 3 Levels
FAST: Functioning Assessment Short Test
FAST-R: Feasibility and Acceptability Support Team for Researchers
GAS: Goal Attainment Scaling
GCP: Good Clinical Practice
GDPR: General Data Protection Regulation
GP: General Practice
HAMA: Hamilton Anxiety Rating Scale
HAMD: Hamilton Rating Scale for Depression
ICC: Intraclass Correlation Coefficient
ICF: Informed Consent Form
IME: Important Medical Events
IQ: Intelligence quotient
ISBD: International Society for Bipolar Disorders
KCL: King’s College London
KCTU: King’s Clinical Trials Unit
MAI: Metacognitive Awareness Inventory
MEQ: Morningness-Eveningness Questionnaire
MINI: Mini International Neuropsychiatric Interview
MoCA-T: Montreal Cognitive Assessment-Telephone version
MSI-BPD: MacLean Screening Instrument for Borderline Personality Disorder
MVAS: Maudsley Visual Analogue Scale
NHS: National Health Service
NIHR: National Institute for Health and Care Research
PANAS-SF: Positive and Negative Affect Schedule
PDQ-D: Perceived Deficits Questionnaire - Depression
PI: Principal Investigator
PIN: Participant Identification Number
PIS: Participant Information Sheet
PrIME: Plan, implement, monitor, and Evaluate
PSQI: Pittsburgh Sleep Quality Index
RCT: Randomised Controlled Trial
REC: Research Ethics Committee
SAE: Serious Adverse Events
SAP: Statistical Analysis Plan
SAR: Serious Adverse Reactions
SD: Standard Deviation
SMART: Specific, Measurable, Attainable, Realistic, and Time-specific
SUAG: Service User Advisory Group
SUSAR: Suspected Unexpected Serious Adverse Reactions
TAU: Treatment as Usual
TMG: Trial Management Group
TMT: Trail Making Test
TOPF: Test of Premorbid Functioning
TSC: Trial Steering Committee
UAR: Unexpected Adverse Reactions
VPA: Verbal Paired Associates
WAI-SR: Working Alliance Inventory-Short Revised
YMRS: Young Mania Rating Scale
